# Association of indoor use of pesticides with CKD of unknown origin

**DOI:** 10.1371/journal.pone.0277151

**Published:** 2023-07-21

**Authors:** Saba Alvand, Sudabeh Alatab, Sahar Dalvand, Fariba Shahraki-Sanavi, Mahmoud Ali Kaykhaei, Elham Shahraki, Erfaneh Barar, Sadaf G. Sepanlou, Alireza Ansari-Moghaddam

**Affiliations:** 1 Liver and Pancreatobiliary Diseases Research Center, Digestive Disease Research Institute, Shariati Hospital, Tehran University of Medical Sciences, Tehran, Iran; 2 Digestive Disease Research Center, Digestive Disease Research Institute, Tehran University of Medical Sciences, Tehran, Iran; 3 Health Promotion Research Center, Zahedan University of Medical Science, Zahedan, Iran; 4 Genetics of Non-Communicable Disease Research Center, Zahedan University of Medical Science, Zahedan, Iran; University of Ruhuna Faculty of Science, SRI LANKA

## Abstract

**Introduction:**

Chronic kidney disease (CKD) is a growing global health problem. Recently, an epidemic of CKD of unknown origin (CKDu), a form of CKD seen mostly in agricultural communities, has been emerged. One of the proposed causes of CKDu is pesticide use in farmers. On the other hand, the research on relation between indoor use of pesticides and CKDu is little. In this study, we aimed to investigate the association between indoor use of pesticide as well as the exposure time with CKDu. This study was done as part of the population-based cohort of Prospective Epidemiological Research Studies in Iran. We used the baseline data of the Zahedan Adult Cohort Study. All subjects with diabetes mellitus and/or hypertension, estimated glomerular filtration rate (eGFR) between 60–89 ml/min/1.73 m^2^, and unavailable creatinine measurement were excluded. Subjects with an eGFR of less than 60 ml/min/1.73 m^2^ were defined as having CKDu, and their data were compared with those with an eGFR of more than 90 ml/min/1.73 m^2^. Data regarding indoor pesticide use and duration of exposure were obtained through a questionnaire. After applying the exclusion criteria, 1079 subjects remained in the study. Female sex, single marital status, low physical activity, triglyceride (TG) levels of more than 150 mg/dl, body mass index (BMI) of more than 25 kg/m^2^, non-smokers, indoor pesticide use, and high pesticide exposure time were associated with CKDu. The effects of age, female sex, TG levels more than 150 mg/dl, pesticide use (OR 1.36; 95% CI 1.01–1.84), and high exposure time (third tertile of exposure time) compared to non-users (OR 1.64; 95% CI 1.07–2.51) remained significant in multivariable analysis.

**Conclusion:**

We found a positive association between pesticide use, as well as longer exposure time to pesticides, and impaired kidney function in cases without diabetes mellitus and hypertension. Further longitudinal studies should be carried out to confirm these findings.

## Introduction

Chronic kidney disease (CKD) as a non-communicable disease has placed a high economic burden on communities to provide high-cost therapies, kidney transplantation, and dialysis, for patients at end stage renal disease (ESRD). Moreover, CKD is a recognized independent risk factor for cardiovascular disease events, which is the main reason for death in these patients [1]. Based on the most recent analysis of the Global Burden of Disease study in 2017, it is estimated that more than 700 million individuals worldwide are affected by CKD. In 2017 alone, over one million people died from CKD [2]. The number of disability-adjusted life-years (DALYs) attributed to CKD reached a significant 35.8 million in 2017 [2]. On the other hand, published data have shown that while the annual change in CKD prevalence in developed countries shows a decreasing trend, the trend is increasing in developing nations, resulting in a significant social and financial burden on the healthcare systems of these countries [2].

Diabetes mellitus type 2 (T2DM) and hypertension (HTN) are the two main etiologies of CKD [3]. Up to 34% of CKD DALYs are due to diabetes mellitus alone [2]. However, in the last 20 years, a new term “CKD of unknown origin (CKDu)” has been introduced in areas of Central America and South Asia that address the diagnosis of CKD which is not attributable to any traditional risk factor, such as diabetes mellitus, hypertension, glomerular nephritis, obstructive nephropathy or congenital structural abnormalities [4–6]. The term CKDu was first used in El Salvador in the early 2000s to describe a disease predominantly affecting agricultural communities. From then, the CKDu is being reported with increasing frequency from predominantly rural locations in several regions across the world [4].

Many hypotheses have been proposed for assessment of potential causes of CKDu. Chronic dehydration and agrochemicals (pesticides, herbicides, fertilizers) use were the most frequent ones assessed in several studies because CKDu is mainly seen in rural men with agricultural occupation [5–7].

However, other environmental factors, such as exposure to heavy metals, high seasonal temperatures, mycotoxins, contaminated water supplies, and snake bites, have also been considered [7–10]. Data regarding the prevalence and epidemiological features of CKDu, especially in developing countries, are scant. CKDu is progressive and often asymptomatic until the late stages, leading to an increasing demand on the healthcare system [11]. Therefore, studies that can help delineate the epidemiological features of this disease or identify contributing causes or risk factors are of great interest.

Sistan and Baluchestan province, with its capital in Zahedan, is the second-largest province located in the southeast of Iran. It is one of the driest regions in the country and has distinct features compared to other provinces, including low population density (52 persons per km^2^), a hot desert climate, a low human development index (HDI) compared to other provinces [12], and a different lifestyle [13]. Although agricultural occupations are scarce in this region, the use of pesticides in yards and homes is more common compared to other parts of Iran [14]. Given these characteristics, this region seems to be a suitable location to investigate the prevalence of CKDu and assess its possible determinants.

In the current study, our aim was to estimate the prevalence of CKDu and assess its main contributors using cross-sectional data from the Zahedan Cohort Study (ZACS), one of the centers of the "Prospective Epidemiological Research Studies in Iran (PERSIAN)" cohort. The PERSIAN cohort is a nationwide cohort study that collects data from subjects aged 35–70 years from 18 cohort centers in 16 provinces of Iran. Detailed information on the PERSIAN Cohort Study can be found elsewhere [5]. ZACS, which is a branch of the PERSIAN cohort, was conducted in three districts of Sistan and Baluchestan with different socioeconomic statuses (low, middle, and high) [16].

## Methods and materials

### Study design and location

**Fig 1:**
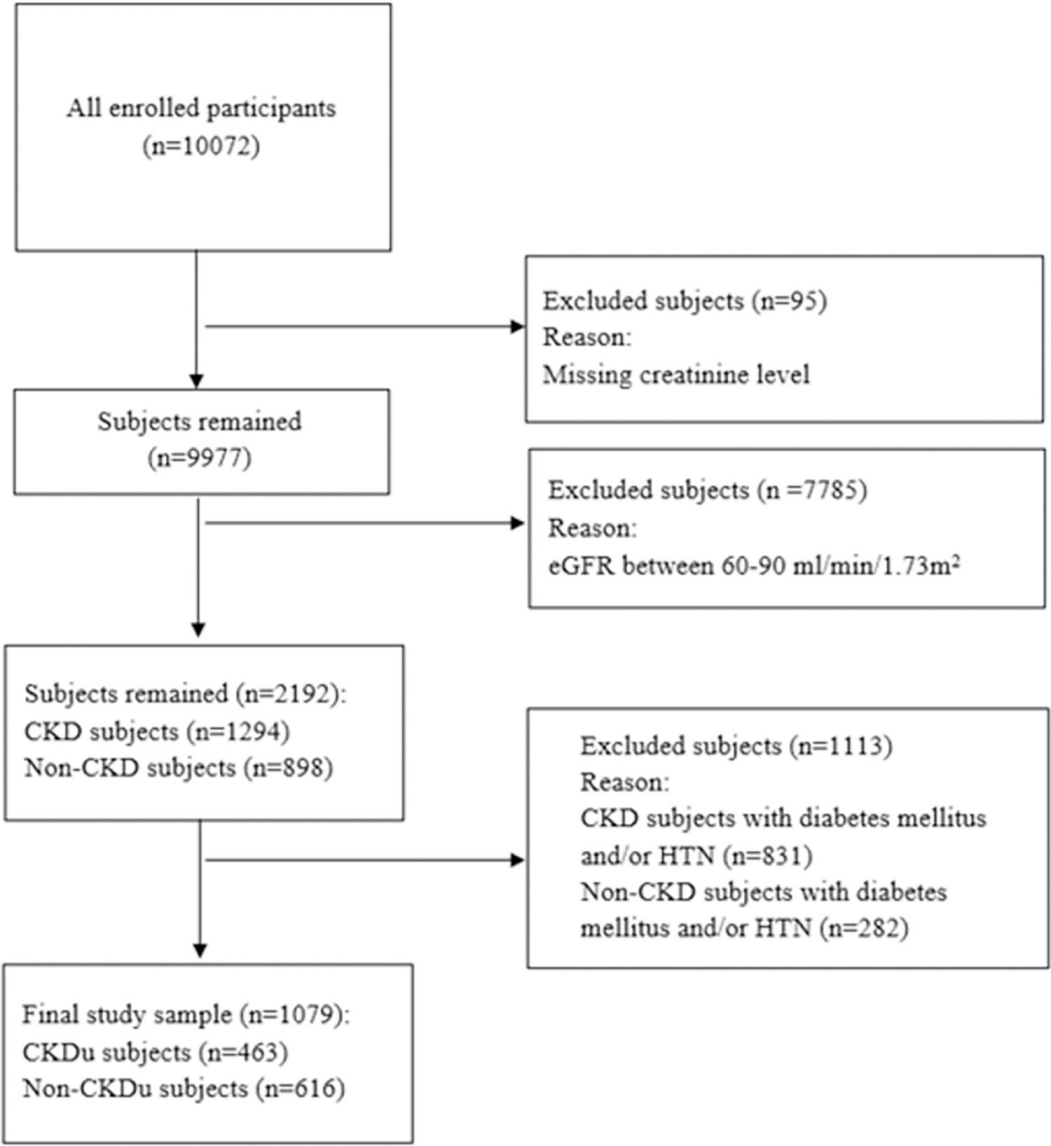
Study sample flow diagram.

This study was a cross-sectional analysis of data from ZACS as part of PERSIAN project. The detailed explanation of PERSIAN study and ZACS has been published previously [15, 16]. In brief, from October 2015 to January 2019, 10,079 subjects who aged 35–70 years and resided for at least nine months in these regions (based on the regional records) enrolled in ZACS study. Subjects with physical or psychological disabilities who could not complete the process were excluded. The Completed flow diagram of study is presented in Fig 1.

Written informed consent was obtained from all participants before enrollment. This study was approved by the ethics committee of Zahedan University of Medical Sciences with the approval number of IR.ZAUMS.REC.1401.160.

The data were collected by employing questionnaires through interviews. The PERSIAN cohort questionnaires consist of three parts: general, medical, and nutrition, and each part contain different topics. The complete data dictionary is available on the PERSIAN cohort site (https://persiancohort.com/). Personal information, anthropometry, physical activity, indoor use of pesticide and duration of exposure were collected from the general section. Past medical history, complete list of medications, and personal habits, were gathered from the medical section. The anthropometrics were obtained in the morning after collecting samples when the participants were still fasting. All the centers used similar adjusted tools for the measurements. Weight was measured by Seca 762 mechanical flat scale in kilograms, and other anthropometrics were measured by Seca 206 body meter measuring tape in centimeters. The trained personnels measured blood pressure with Riester auscultatory sphygmomanometers based on the American heart association criteria [15, 17].

Blood sample was collected from all participants after 8–12 h of fasting. The blood samples were transferred to the PERSIAN cohort reference laboratory within 3 h of sampling for measuring complete blood count, fasting blood sugar (FBS), total cholesterol, high-density lipoprotein cholesterol (HDL), triglycerides (TG), alanine transaminase (ALT), aspartate transaminase (AST), alkaline phosphatase (AlkP), gamma-glutamyl transpeptidase, blood urea nitrogen (BUN), and creatinine levels.

### Definitions

HTN was defined as having any of the following conditions: self-report use of hypertension medications, anti-hypertensive medication consumption, systolic blood pressure (SBP) ≥ 140 mmHg, or diastolic blood pressure (DBP) ≥ 90 mmHg [18].

Diabetes Mellitus was defined as having any of the following conditions: self-report use of diabetes mellitus medications, blood glucose-lowering medications consumption, FBS ≥ 126 mg/dl [19].

Physical activity status was included in the analysis as tertiles using metabolic equivalent task (MET) rates of self-reported daily activities of subjects obtained through a questionnaire [20]. Based on the mean MET rates of the study subjects, they were classified into three groups: 1st tertile (≤35.3 METs/hour/day), 2nd tertile (35.4–38.9 METs/hour/day), and 3rd tertile (≥39 METs/hour/day).

Hypertriglyceridemia (Hyper TG) was defined as serum triglyceride (TG) levels ≥150 mg/dl. Low high-density lipoprotein (HDL) was defined as serum HDL levels <40 mg/dl for men and <50 mg/dl for women.

Pesticide exposure was evaluated based on the questionnaire asking about the ever use, frequency and duration of exposure to pesticide(s). In case of a positive exposure (yes/ no answer), the frequency and duration of exposure were asked. The sum of frequency and exposure time was calculated and expressed in minutes. The duration of pesticides exposure in lifetime was treated as a continuous variable and included in the analysis in three groups of less than 9 minutes (1^st^ tertile), between 9–20 minutes (2^nd^ tertile) and more than 20 minutes (3^rd^ tertile).

The standard colorimetric Jaffe-Kinetic reaction method were used to measure serum creatinine levels, which was not traceable to isotope dilution mass spectroscopy (IDMS). Since we did not have access to albuminuria, the kidney damage was investigated by estimated glomerular filtration rate (eGFR) measurement alone. MDRD study equation was applied for estimating GFR based on the following formula:

GFR by MDRD (ml/min/1.73 m^2^)  =  176 × Cr^-1.154^ × age^-0.203^ × 0.742 (if female)

GFR by MDRD (ml/min/1.73 m^2^)  =  176 × Cr^-1.154^ × age^-0.203^ × 1(if male)

CKD was based on one measurement of serum creatinine and defined as participants who had eGFR less than 60 ml/min/1.73 m^2^ [21, 22]. Participants with eGFR higher than 90 ml/min/1.73 m^2^ were counted as normal eGFR due to unclear kidney damage in this spectrum without urine data [23].

CKDu group defined as subjects who had eGFR less than 60 ml/min/1.73 m^2^ without diabetes mellitus and HTN (based on the mentioned definitions). Non-CKDu group defined as subjects with eGFR higher than 90 ml/min/1.73 m^2^ without diabetes and HTN. Since this study is nested from a large population-based cohort, exclusion of other known etiologies for CKDu through ultra-sonography (to exclude structural kidney diseases) and urine analysis (to exclude glomerular diseases) were not cost-benefit and applicable.

### Statistical analysis

Frequency and percentage were used to describe qualitative variables, and a comparison of CKDu and non-CKDu groups was assessed through a Chi-squared test. TG and HDL were used as binary variables. Mean and standard deviation were used to describe quantitative variables, and a student t-test was applied to check the difference between the two main groups of the study.

In this study, we used regression analysis to identify associations, determine the magnitude of the relationship, and assess the significance between the study variables and our outcome (CKDu). The unadjusted association between variables and the outcome (CKDu) was evaluated using univariable logistic regression analysis. Variables with p-values less than 0.20 in the univariable analysis were included for multivariable logistic regression analysis using two models. Model 1 included the following variables: age, sex, marital status, BMI, smoking, hypertriglyceridemia (hyper TG), and indoor use of pesticides. In model 2, we added the duration of pesticide exposure to the other included variables. Statistical significance was declared if the p-value was less than 0.05, and all analyses were performed using STATA (version 12; StataCorp).

## Result

From 10,072 subjects who participated in ZACS, 95 subjects did not have blood samples and therefore were excluded from the study (Fig 1). The mean age of participants was 50.4 years (SD = 9.2), and 39.1% were male. The prevalence of CKD in our sample was 9.1% (95% confidence interval (CI): 8.6–9.6) which was 5.0% (95% CI: 4.4–5.7) in males and 13.3% in females (95% CI: 12.4–14.1).

In our population, 7,785 (78%) subjects had eGFR between 60–89 ml/min/1.73m^2^ and therefore excluded from our analysis. Among the remaining 2,192 subjects, the prevalence of CKD was 59.03% (n = 1294). The prevalence of subjects with diabetes mellitus and/ or HTN was 64.2% in our CKD group and 31.4% in non-CKD group (Fig 1).

Among the 1,079 remaining subjects, 463 subjects (42.9%) had eGFR less than 60 ml/min/1.73m^2^ (CKDu group) and 616 participants had eGFR higher than 90 ml/min/1.73m^2^ (non-CKDu group). All CKDu subjects were in stage 3.

The mean age of subjects in CKDu group was significantly higher than non-CKDu group (52.1 ± 9.1 years vs. 45.6 ± 8.2; p = 0.02). The male subjects comprise 61.4% of non-CKDu, while in CKDu this percentage was significantly lower (20.7%, (p<0.001). The number of subjects who did not use pesticides were significantly lower in CKDu compared to the non-CKDu group (49.7% vs 59.3%, p = 0.002). Accordingly, subjects in CKDu group had significantly higher pesticides exposure time compared to non-CKDu subjects (p = 0.01). Table 1 demonstrates the details of the demographics and medical history of the included subjects in two groups.

**Table 1:** Demographics, medical history, and house use of pesticides in the studied population.

Parameters		Non-CKDu (n = 616)	CKDu (n = 463)	*P*-value
Age, mean (SD) (years)		45.58 ± 8.21	52.07 ± 9.06	0.022
Sex, n (%)	Male	378 (61.36)	96 (20.73)	<0.001
	Female	328 (38.64)	367 (79.27)	
Marital status, n (%)	Single	46 (7.47)	92 (19.87)	<0.001
	Married	570 (92.53)	371 (80.13)	
Physical activity, n (%)	1^st^Tertile (< = 35.3)	209 (33.93)	151 (32.61)	0.025
	2^nd^ Tertile (35.4–38.9)	186 (30.19)	174 (37.58)	
	3^rd^Tertile (> = 39)	221 (35.88)	138 (29.81)	
Low HDL n (%)	No	319 (51.79)	221 (47.73)	0.187
	Yes	297 (48.21)	242 (52.27)	
Hyper TG n (%)	No	458 (74.35)	290 (62.63)	<0.001
	Yes	158 (25.65)	173 (37.37)	
BMI n (%)	<18.5	68 (11.07)	10 (2.16)	<0.001
	18.5–25	257 (41.86)	140 (30.24)	
	> = 25	289 (47.07)	313 (67.60)	
Smoking status n (%)	Non smoker	446 (72.40)	412 (88.98)	<0.001
	Current smoker	64 (10.39)	19 (4.10)	
	Previous smoker	106 (17.21)	32 (6.91)	
Indoor use of pesticides n (%)	No	365 (59.25)	230 (49.68)	0.002
	Yes	251 (40.75)	233 (50.32)	
Duration of exposure to indoor pesticides (minutes) n (%)	Non users	365 (59.25)	230 (49.68)	0.011
	1^st^ Tertile (<9)	83 (13.47)	82 (17.71)	
	2^nd^ Tertile (9–20)	90 (14.61)	72 (15.55)	
	3^rd^ Tertile (>20)	78 (12.66)	79 (17.06)	

In univariate analysis, female gender had the greatest crude odds ratio for CKDu (6.07, 95% CI 4.60–8.01). The effect of gender was raised in both multivariate models, compared to univariate analysis, reaching an odd of 7.52 (95% CI: 5.69–12.77) in model 1 and 8.64 (95% CI: 5.76–12.98) in model 2. Notably, the single marital status that had the second greatest odds ratio following female gender in univariate analysis (OR: 3.07, 95% CI: 2.11–4.48), was not associated with CKDu in multivariate analysis. Our analysis showed that age, hyper TG and BMI were among determinants of CKDu that kept their association in both models.

The indoor use of pesticides showed a significant association with CKDu in univariable analysis with an odds ratio of 1.47 (95% CI: 1.15–1.87); the effect was present in model 1. Duration of exposure to indoor pesticides had a significant association in univariable analysis; however, only the highest exposure time (3^rd^ tertile) had a significant association in model 2, in a way that subjects in this category showed 64% higher odds of CKDu than non-users.

## Discussion

The indoor use of pesticides is a common practice, but the kidney health outcomes associated with this practice have received less attention in exposure-related research. In this perspective, the purpose of this study is innovative.

The prevalence of CKD in this region in our study was 9.1% (95% CI 8.6–9.6) based on the MDRD equation. This figure was slightly lower than the findings of the Tehran Lipid Glucose Study (TLGS) [24]. The main reason might be the higher percentage of male subjects included in the TLGS study, as many studies have shown a higher prevalence of CKD among women compared to men [23, 25–29]. In another study conducted in the Khuzestan region (southwest of Iran), the prevalence of CKD was reported at 7.1% [23], which was slightly lower than what we found in the current study (9.1%). The younger age spectrum in the Khuzestan study might partially explain the difference in CKD prevalence between the two studies. On the other hand, Sepanlou and colleagues reported a high prevalence of CKD (total: 23.7%, women: 26.6%, men: 20.6%) in subjects older than 40 years old using data from the Golestan cohort study (a region in northeast Iran) [28].While we cannot completely explain this variation, but difference in ethnicities could be among the reasons considering that more than three-quarters of the GCS population were Turkmen, while Sistan and Baluchestan population comprise of different ethnicities.

We found that in our study, prevalence of CKD in females was 2.6 times higher than male subjects, a proportion that is higher than other areas of our country and even other countries [23, 25–29]. For instance, a systematic review of CKD epidemiological features in Iran reported that prevalence of CKD in females was 1.7 times higher than in males, a figure that was much lower than our findings [30]. The odds of having CKDu raised to 7.5 and 8.6 times in women when we analyzed it in our multivariable models including indoor use of pesticides (model 1) and duration of exposure to indoor pesticides (model 2), respectively. This finding might point to risk of pesticides use as women spent more time at home in this area and subsequently, they might have an increased risk of pesticide exposure.

In our study, the duration of exposure to indoor use of pesticides was significantly higher in subjects in the CKDu group compared to those in the non-CKDu group (50.3% vs. 40.8%). This difference remained significant even after adjusting for other variables in multivariable model 1. Subjects with a history of indoor pesticide use had 1.36 times higher odds of CKDu compared to their counterparts (Table 2). In Iran, low doses of organophosphates have been used in many insecticides, and handmade insecticides and pesticides are not uncommon in low-income families. Trabanino and colleagues described CKDu for the first time in 2002; they found out most of these patients were farmers who had been exposed to pesticides regularly [31]. In 2014, after many studies observed the impairment of kidney function among farmers, a study was designed on US Agricultural Health Study to determine the exposure-response trends among licensed pesticide applicators. They reported positive exposure trends in use of herbicides alachlor, atrazine, metolachlor, paraquat, pendimethalin, and the insecticide permethrin in subjects with ESRD [32]. Farmer’s wives were at risk of pesticides, which suggests that small amounts of pesticides and indirect exposure can also be harmful [32]. Jayatilake and colleagues found that the chronic low dose of cadmium exposure can result in CKD of unknown origin, and co-exposure to arsenic has an additive effect on impairment of kidney function [33]. However, in a meta-analysis in 2018 in Meso-America, there was no association between pesticides exposure and GFR <60 mL/min/1.73 m2 as well [34]. Since the chemical base of insecticides and pesticides are similar and pesticides may be used at home too, there is an urge to clear the effect of house use of pesticides and insecticides on kidney function.

**Table 2:** Risk factors for CKD of unknown origin in univariable and multivariable logistics regression analysis.

		Univariable model		Multivariable model			
				Model 1 ^§^		Model 2 ^§§^	
**Variable**		OR	95% CI	OR	95% CI	OR	95% CI
Age		1.08	1.07–1.10	1.11	1.09–1.13	1.1	1.09–1.13
Sex	Male	Ref		Ref		Ref	
	Female	6.07	4.60–8.01	7.52	5.69–12.77	8.64	5.76–12.98
Marital status	Married	Ref		Ref		Ref	
	Single	3.07	2.11–4.48	1.50	0.93–2.42	1.50	0.93–2.42
Physical activity	1^st^ Tertile	1.15	0.85–1.55	-		-	
	2^nd^ Tertile	1.49	1.11–2.01	-		-	
	3^rd^ Tertile	Ref		-		-	
*P for linear trend*		0.338		-		-	
Low HDL	No	Ref		-		-	
	Yes	1.17	0.92–1.49	-		-	
Hyper TG	No	Ref		Ref		Ref	
	Yes	1.72	1.33–2.24	1.53	1.11–2.12	1.55	1.12–2.15
BMI	<18.5	0.26	0.13–0.54	0.32	0.14–0.72	0.32	0.14–0.73
	18.5–25	Ref		Ref		Ref	
	> = 25	1.98	1.53–2.58	1.33	0.96–1.84	1.32	0.95–1.84
*P for linear trend*		<0.001		<0.001		<0.001	
Smoking status	Non smoker	Ref		Ref		Ref	
	Current smoker	0.32	0.18–0.54	1.04	0.53–2.05	1.05	0.53–2.07
	Previous smoker	0.32	0.21–0.49	0.97	0.55–1.70	1.00	0.57–1.74
Indoor use of pesticides	No	Ref		Ref		-	
	Yes	1.47	1.15–1.87	1.36	1.01–1.84	-	
Duration of exposure to indoor pesticides (minutes)	Non users	Ref		-		Ref	
	1^st^ Tertile (<9)	1.56	1.10–2.21	-		1.33	0.87–2.03
	2^nd^Tertile (9–20)	1.26	0.89–1.80	-		1.16	0.75–1.79
	3^rd^Tertile (>20)	1.60	1.12–2.28	-		1.64	1.07–2.51
*P for linear trend*		0.038				0.056	

^§^ Model 1 included variables: age, sex, marital status, BMI, smoking, TG, house use of pesticides

^**§§**^ Model 2 included variables: age, sex, marital status, BMI, smoking, TG, house use of pesticides exposure time

We found a positive relationship between the duration of exposure to indoor pesticide use and CKDu. Subjects categorized in the highest tertile of exposure duration to indoor pesticide use had 1.64 times higher odds of having CKDu compared to never users. This finding emphasizes the role of cumulative exposure dose at a specific time on kidney function. Although we cannot comment on safe threshold dose of house use of pesticides, as this was not in our study scope, but finding the safe use threshold of these materials could be of great interest that could be evaluated in longitudinal studies.

We must emphasize that the relationship between pesticide use (both usage and duration) and CKDu, as obtained in this study, should be interpreted cautiously. It is important to note that we lacked data on outdoor pesticide exposure, such as occupational practices. This limitation should be taken into consideration when assessing the relationship between nephropathy and pesticides. The prevalence of CKD excluding subjects with HTN and / or diabetes mellitus was approximately 36% in our study, while it was much lower in studies in Malawi (0.2%) [35] and India (1.6%) [36]. Even though participants with proteinuria were excluded from the mentioned studies, our finding was more than expected.

A low HDL did not have an association with CKDu in this study. Low level of HDL is commonly seen in the end stages of CKD due to impairment of reverse cholesterol transport [37]. We should emphasis that all our subjects in CKDu group had CKD stage 3. This might explain why we could not see a significant effect of low HDL level on CKDu. Ghosh and colleagues demonstrated positive association of total lipid level with CKDu [38]. The total lipid was calculated by sum of 2.27 times of total cholesterol, triglycerides plus 0.623 and did not address HDL and triglycerides separately. We also found that higher TG levels have a significant relation with CKDu, an effect that remained even in multivariable regression. The relation between dyslipidemia and CKD has been proposed by many studies [25, 39–42]. Studies showed that higher level of TG is seen in the early stages of CKD [43]. A multicenter cohort study of non-dialysis dependent outpatients with CKD in Japan reported a decreasing effect of high TG with CKD staging as well [32]. Taken together, this might justify the significant odds of high TG levels on CKDu, as all the participants categorized in CKDu had CKD stage3.

One limitation of this study is that since this study is part of the national cohort study, urine sampling and kidney sonography was not cost beneficial and were not performed. Thus, not all the CKDu patients had unknown etiology. However, we tried to minimize the known CKD patients with accurate screening for diabetes mellitus and HTN based on the PERSIAN cohort protocol. Another limitation of this study was a one-time measurement of serum creatinine, which can be misleading because of variation due to weather, season, and work pattern; thus, our finding must be confirmed by future studies with at least two times measurement of creatinine. Although epidemiologic studies have internal limitations in assessing CKD and its etiologies, they are the first step in assessing risk factors and protective factors and should not be overlooked. Using novel biomarkers like KIM-1 and NGAL can facilitate the estimation of kidney damage in national epidemiologic studies as well [43]. Another limitation of this study is the lack of information regarding the specific types of pesticides used, the amount and frequency of pesticide usage, as well as the purpose of their application.

## Conclusion

In this study, we conducted a population-based study in a specific region of our country to assess the prevalence of CKDu and identify possible determinants. We found that indoor use of pesticides could be a potential risk factor for CKDu. Previous research has highlighted the potential harm of pesticides on kidney function, particularly in outdoor uses. Our findings raise concerns about the impact of indoor pesticide use on kidney function in individuals without common risk factors for CKD. Further longitudinal studies are needed to evaluate the effects of indoor pesticide use on kidney health outcomes and to determine safe dosage levels for these substances.
